# Lobectomy for lung cancer in a myelodysplastic syndrome patient with decreasing platelet aggregation: report of a case

**DOI:** 10.1186/s13019-018-0777-7

**Published:** 2018-07-24

**Authors:** Satoshi Koezuka, Yoshinobu Hata, Hajime Otsuka, Takashi Makino, Yoko Azuma, Takashi Azumi, Yoichi Anami, Kazuhiko Natori, Akira Iyoda

**Affiliations:** 1 Division of Chest Surgery, Toho University School of Medicine, Tokyo, Japan; 2 Division of Hematology and Oncology, Toho University Medical Center, Tokyo, Japan

**Keywords:** Myelodysplastic syndrome, Lung cancer, Platelet aggregation

## Abstract

**Background:**

Myelodysplastic syndromes (MDS) are clonal stem cell disorders of the bone marrow. Most patients with MDS have a high risk of bleeding. Thrombocytopenia and defective platelet aggregation contribute to bleeding. We report a surgical case of a patient with lung cancer concomitant with MDS.

**Case presentation:**

A 72-year-old man presented to our hospital because of an abnormal shadow on chest x-ray suggesting a primary lung cancer. A peripheral blood smear examination found giant platelets without thrombocytopenia. He was diagnosed with MDS by bone marrow biopsy, and showed defective platelet aggregation despite a normal bleeding time. The patient underwent left lower lobectomy and transfusion of platelets because of chest wall bleeding.

**Conclusions:**

We demonstrated that ordering platelet preparations might be desirable for an MDS patient with defective platelet aggregation who will undergo surgery, even for a normal platelet count and bleeding time.

## Background

Myelodysplastic syndromes (MDS) are clonal stem cell disorders of the bone marrow, which are characterized by ineffective hematopoiesis and morphological and functional abnormalities of hematopoietic cells [1]. The annual disease incidence is about four per 100,000 people, and the incidence increases with age [2]. The risk of cancer developing in patients with MDS has been found to be 2.9-fold higher than the expected rate [3]. Thrombocytopenia and neutropenia are seen at diagnosis in about a third of patients, and anemia is also frequently observed [1]. Therefore, most patients with MDS have a high risk of infection or bleeding. To our knowledge, there has only been one case report of a lung cancer patient with MDS who underwent surgery in the English literature [4]. Here, we report a surgical case of a patient with lung cancer concomitant with MDS. The patient’s platelet count and bleeding time were normal, but he had defective platelet aggregation, which resulted in bleeding at the time of surgery.

## Case presentation

**Fig. 1:**
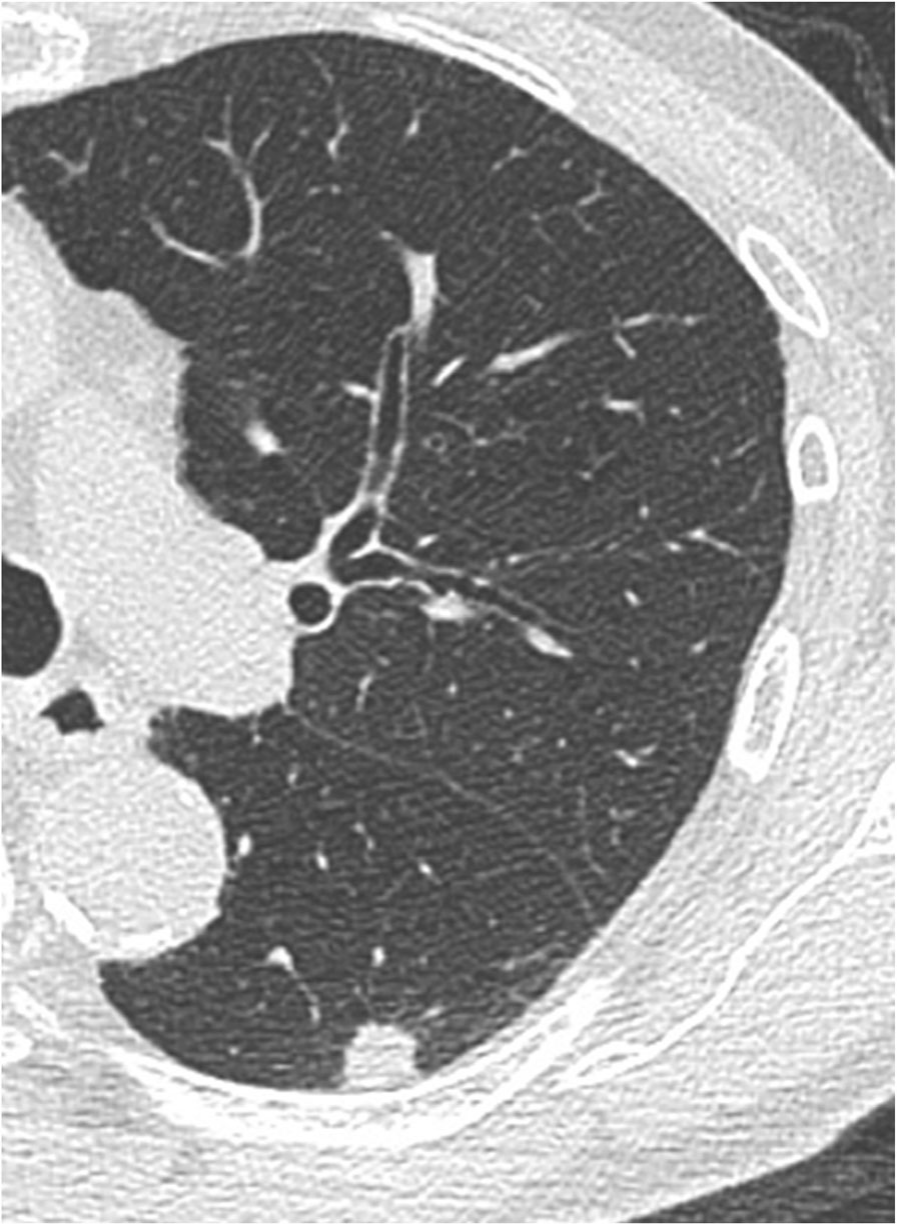
Chest computed tomography image showing a solid nodule in the left lower lobe

**Fig. 2:**
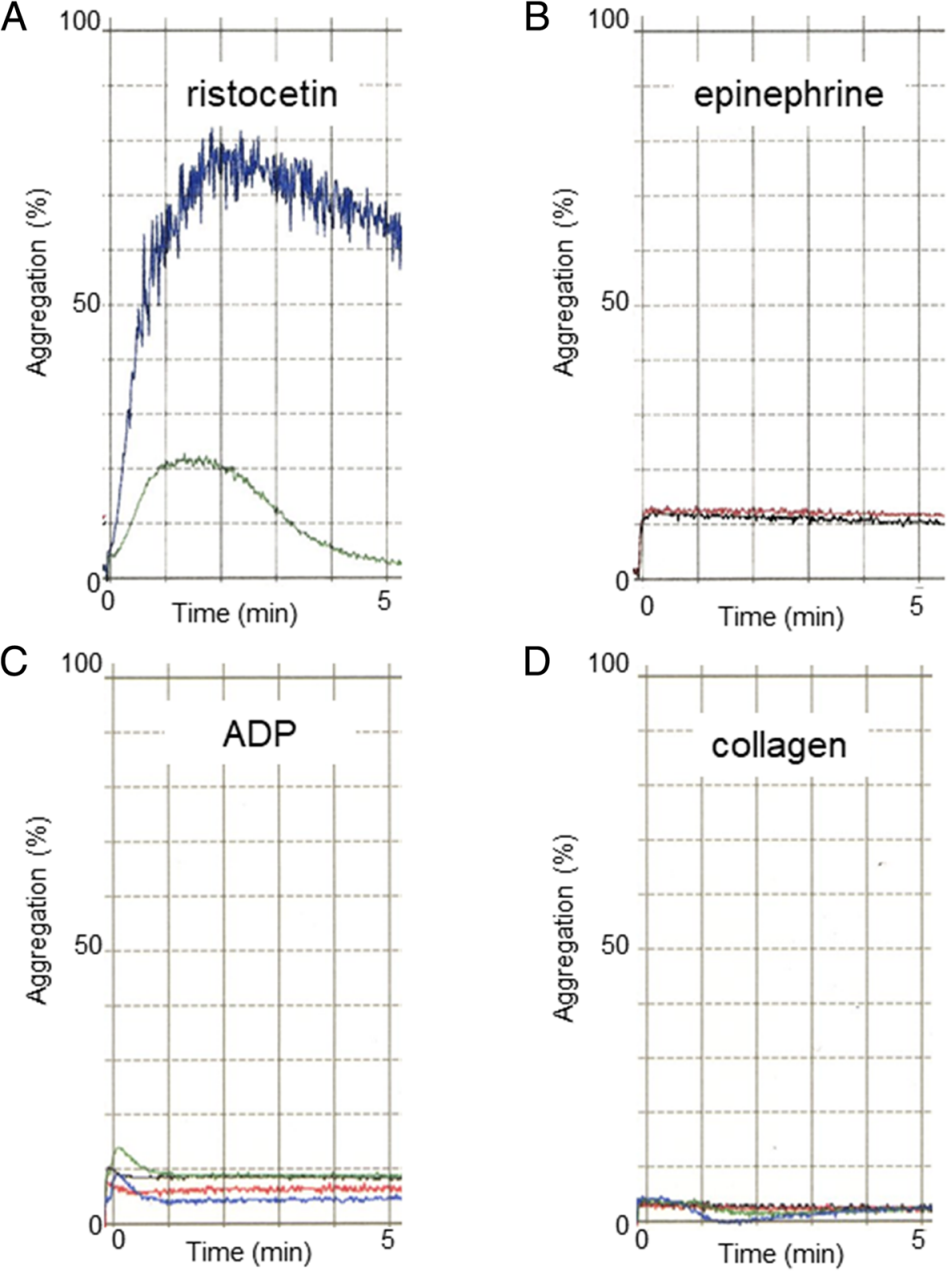
Platelet aggregation tests. **a** Platelet aggregation with ristocetin was normal. **b**–**d** Platelet aggregation with epinephrine, ADP, and collagen was severely decreased

A 72-year-old man presented with cough. He was referred to our hospital because of an abnormal shadow found on a chest x-ray. His medical history included chronic obstructive pulmonary disease and hyperlipidemia. Chest computed tomography revealed a 12-mm solid nodule in the left lower pulmonary lobe without notable mediastinal lymph node enlargement (Fig. 1). 18-Fluorodeoxyglucose-positron emission tomography/computed tomography showed a nodule in the left lower lobe with an increased uptake (maximum standardized uptake value of 5.3). Contrast-enhanced magnetic resonance imaging of the head was negative for metastasis. A primary lung cancer (cT1bN0M0-stageIA2) was suspected; and the patient underwent bronchoscopy, which did not produce a definitive diagnosis. Presurgical evaluation of cardiac function revealed an electrocardiographic abnormality. Coronary angiography showed moderate stenosis of the left anterior descending artery, but lobectomy was judged possible. Laboratory testing found a white blood cell count of 2600/μL with 16% neutrophils; hemoglobin, 11.5 g/dL; and platelet count, 155,000/μL; along with giant platelets in a blood smear, which suggested a hematologic disease. A bone marrow biopsy led to a diagnosis of MDS. The bone marrow biopsy revealed normocellular with 3.2% blasts. The chromosome study showed a 46,XY,+1,der(1;7)(q10;p10). The patient’s bleeding time, prothrombin time, and activated partial thromboplastin time were in the normal range. Platelet dysfunction was a concern because of the giant platelets, and platelet aggregation was tested by the agonists ristocetin, epinephrine, adenosine diphosphate (ADP), and collagen before surgery. Ristocetin-induced platelet aggregation was normal, but epinephrine-, ADP-, and collagen-induced platelet aggregation was severely decreased (Fig. 2a–d). We ordered platelet preparations for possible intraoperative bleeding. The patient underwent left lower lobectomy and subsequent lymph node sampling by video-assisted thoracoscopy. Intraoperative examination of the frozen section revealed non-small cell lung cancer. The surgery initially was performed without bleeding or other complications; however, just before the chest was closed, oozing from the intercostal muscles was observed, and hemostasis by electrocoagulation could not be obtained. The patient received 20 units of platelet concentrates, and bleeding from the intercostal muscles was controlled thereafter. On histopathology, tumor cells with a very big nucleus were forming a fuller nest and proliferating. Immunostaining of the tumor cells was positive for p40 and CK5/6, but negative for napsin A and TTF-1. The pathological diagnosis of the tumor was poorly differentiated squamous cell carcinoma. The patient’s postoperative course was uneventful and without bleeding complications. He was discharged on postoperative day 10. Two years after surgery, the patient is alive without any signs of lung cancer recurrence.

## Discussion

Prognosis of MDS is directly related to the number of bone marrow blast cells, to certain cytogenetic abnormalities, and to the amount of peripheral blood cytopenias. The most widely used prognostic classification system used for the MDS is the International Prognostic Scoring System (IPSS) [5]. This case is a lower risk in the IPSS classification which many patients die from causes other than MDS, and the median survival period is 3.5 years [1]. Supportive care is the cornerstone of all MDS treatments and prevent symptoms from low blood counts. Surgery for MDS patients is not often done because of the risk of infectious, bleeding and anemia. In this case, stage IA lung cancer was suspected, surgery could be expected to be radical, and chemotherapy was considered to be difficult because of MDS. Therefore, we underwent surgery.

MDS is often associated with spontaneous life-threatening bleeding. The prevalence of bleeding reported in the literature has ranged from 3 to 53% [6]. In addition to thrombocytopenia, defective platelet aggregation contributes to bleeding. However, little is known about the defective platelet aggregation of MDS. A study by Zeidman et al. found that 16 of 24 patients (70%) with MDS had defective platelet aggregation [7]. We believe that even without thrombocytopenia, platelet aggregation tests should be performed before surgery to predict bleeding risk in MDS patients.

As seen for our case, defective platelet aggregation causes clinical bleeding, even in patients with a normal platelet count and bleeding time. Neukirchen et al. reported that in patients with platelets >50,000/μL, they found signs of bleeding which might have been attributable to platelet dysfunction in 19% of patients [8]. Zeidman et al. reported that they performed platelet function studies on selected MDS patients with platelet counts >70,000/μL. Six of their 23 patients (26%) presented with signs of bleeding, and five of these six patients showed at least one agonist that induced defective platelet aggregation on platelet aggregometry, despite a normal bleeding time [7].

Platelet aggregometry assesses the aggregation of platelets in response to various platelet activators (ADP, collagen, epinephrine, ristocetin). In our case, epinephrine-, ADP-, and collagen-induced platelet aggregation was strongly decreased. Most reports could not correlate any type of defective platelet aggregation with clinical bleeding. In general, bleeding time assessment is poorly reproducible and insensitive. Our patient did not manifest bleeding at the time of cardiac catheterization and arterial blood gas testing, but did bleed during surgery. To the best of our knowledge, no reports have been published on surgery for MDS patients with defective platelet aggregation. When we perform surgery on an MDS patient with defective platelet aggregation, we should take into consideration a possible bleeding event regardless of a normal platelet count and bleeding time.

Platelet transfusion is commonly considered for patients who undergo major surgery with a platelet count of 50,000/μL or less; and besides, prophylactic use of platelet transfusions for decreasing platelet aggregation is not indicated [9]. We can not predict how much platelet function agglutination ability will decline and how difficult it will be to stop bleeding during surgery. In this case, just before the chest was closed, oozing from the wide range of intercostal muscles was observed, and hemostasis by the energy device was unable to stop the bleeding. Therefore, we judged that coagulation factor was consumed during surgery and we could stop bleeding after platelet transfusion. Our patient underwent surgery with platelet units ordered, but it was not administered from the start of surgery. Because platelets were consumed during surgery and the half-life of platelets was short, it was thought that administration from the beginning was not efficient. We demonstrated that the preoperative request of platelet preparations might be desirable for an MDS patient with defective platelet aggregation, even if the platelet count and bleeding time are normal.

## Conclusions

We report this case because MDS patients with defective platelet aggregation are at risk for bleeding during surgery, even if the platelet count and bleeding time are normal. A preoperative request for platelet units should be implemented for such cases.

## Abbreviations

ADP: Adenosine diphosphate
MDS: Myelodysplastic syndromes
